# Metabolic dependencies govern microbial syntrophies during methanogenesis in an anaerobic digestion ecosystem

**DOI:** 10.1186/s40168-019-0780-9

**Published:** 2020-02-15

**Authors:** Xinyu Zhu, Stefano Campanaro, Laura Treu, Rekha Seshadri, Natalia Ivanova, Panagiotis G. Kougias, Nikos Kyrpides, Irini Angelidaki

**Affiliations:** 1 Department of Environmental Engineering, Technical University of Denmark, Building 115, DK-2800 Kgs. Lyngby, Denmark; 2 US Department of Energy, Joint Genome Institute, Walnut Creek, CA USA; 3 Department of Biology, University of Padua, Via U. Bassi 58/b, 35121 Padua, Italy; 4 CRIBI Biotechnology Center, University of Padua, 35131 Padua, Italy; 5 Soil and Water Resources Institute, Hellenic Organisation-DEMETER, 57001, Thermi-, Thessaloniki, Greece

**Keywords:** Anaerobic digestion, Microbial community, Metagenomics, Metatranscriptomics, Auxotrophies, Syntrophic acetate oxidation, Glycine cleavage, Methanogenic pathways

## Abstract

Methanogenesis, a biological process mediated by complex microbial communities, has attracted great attention due to its contribution to global warming and potential in biotechnological applications. The current study unveiled the core microbial methanogenic metabolisms in anaerobic vessel ecosystems by applying combined genome-centric metagenomics and metatranscriptomics. Here, we demonstrate that an enriched natural system, fueled only with acetate, could support a bacteria-dominated microbiota employing a multi-trophic methanogenic process. Moreover, significant changes, in terms of microbial structure and function, were recorded after the system was supplemented with additional H_2_. *Methanosarcina thermophila*, the predominant methanogen prior to H_2_ addition, simultaneously performed acetoclastic, hydrogenotrophic, and methylotrophic methanogenesis. The methanogenic pattern changed after the addition of H_2_, which immediately stimulated *Methanomicrobia*-activity and was followed by a slow enrichment of *Methanobacteria* members. Interestingly, the essential genes involved in the Wood-Ljungdahl pathway were not expressed in bacterial members. The high expression of a glycine cleavage system indicated the activation of alternative metabolic pathways for acetate metabolism, which were reconstructed in the most abundant bacterial genomes. Moreover, as evidenced by predicted auxotrophies, we propose that specific microbes of the community were forming symbiotic relationships, thus reducing the biosynthetic burden of individual members. These results provide new information that will facilitate future microbial ecology studies of interspecies competition and symbiosis in methanogenic niches.

Video abstract.

Video abstract.

## Background

Microbial methanogenic metabolism is considered as one of the oldest bio-activities on earth and draws great attention because of its global warming potential [1] , which is 28 times higher than carbon dioxide (CO_2_) on a 100-year horizon [2]. In a natural ecosystem, around one billion tons of methane (CH_4_) is formed through microbial activity as an intermediate step of the global carbon cycle [3]. Nevertheless, an enhanced and well-controlled methanogenic process has implications for energy generation [4] due to its high calorific value. Microbial methanation was extensively employed in vessel ecosystems, i.e. biogas reactors, to attain large-scale production as a sustainable energy source. It is postulated that methanogenesis is performed mainly through acetoclastic, hydrogenotrophic, and secondary through methylotrophic pathways in oxygen-depleted environments. The known methanogenic members belong mainly to phylum *Euryarchaeota,* with few exceptions, which were recently assigned to candidate phyla “Bathyarchaeota” [5] and “Verstraetearchaeota” [6]. All methanogens are physiologically specialized and able to scavenge the electrons from hydrogen (H_2_), formate, methanol, and acetate, having CH_4_ as the final product. Archaeal growth and activity can create ecological niches for the oxidizing (H_2_ producing) bacteria, and form syntrophic relations in a complex community.

In the past years, genome-centric metagenomics was extensively used to describe complex syntrophic microbial communities, and successfully revealed essential knowledge regarding the microbial functions of the keystone species mainly based on their gene profiles [7, 8] . The majority of studies regarding methanogenic process were focused on specific microbes contributing to the degradation of recalcitrant substrates [9] or the involvement of rare taxon in the methanogenic process [10, 11], while few attempts have been made to underlie the basic mechanisms of microbial

community assembly and function [7, 12, 13]. In natural ecosystems, the holistic untangling of the intricate methanogenic process was hampered by the inextricable influence of numerous environmental variables occurring simultaneously. Moreover, the in-situ activity of the individual members in microbial communities and the ecological relationships existing among microbes were extremely difficult to elucidate during the digestion of complexed substrates. Thus, simplified model systems are required to unveil the fundamental metabolic insights into methanogenic activities. A previous study dissected the complex methanogenic consortium into tractable model sections by substrates specification in continuous reactor operation and successfully assigned putative functional roles to the de-novo reconstructed genomes [12]. However, a crucial limitation of studies based solely on metagenomic surveys is the lack of direct evidence for the activity of individual microbes. Therefore, other –omics approaches and advanced molecular tools, such as transcriptomics, proteomics, metabolomics, and stable isotope labelling were gradually introduced to analyze the microbial activity during the methanogenic process [14–17].

The current study is dedicated to unveil the core microbial methanogenic metabolisms with combined genome-centric metagenomic and metatranscriptomic strategies. The methanogenic metabolism was favoured in microcosms where the microbial communities were simplified by providing a chemically-defined substrate (acetate). The study revealed the in situ activity of methanogens in syntrophic microbial communities and their affinity to H_2_ provision. Moreover, this work also provided mechanistic understandings of the bacterial functionalities both for acetate oxidation and revealed important auxotrophic dependencies, as well as community structure maintenance during methanogenesis.

## Materials and methods

### Experimental set-up

The triplicate lab-scale biogas continuous stirred-tank reactors (working volume 1.8L) were inoculated with digestate from full-scale thermophilic biogas plant (Snertinge, Denmark). The plant was fed with 70–90% animal manure and 10–30% food industrial organic waste; therefore, the inoculum provided the microbial community to adopt to heterogeneous substrate degradation. During the experiment, the reactors were fed with synthetic medium, in which only acetic acid was supplied as an organic carbon source. Other nutrients were provided by basal anaerobic medium [18]. The reactors operated under thermophilic condition (55 °C) and the operational parameters were chosen according to empirical experiences of highly efficient thermophilic biogas reactors, i.e. the organic loading rate was 1g acetic acid/day. L-reactor and the hydraulic retention time was 15 days. The reactors were fed four times per day with peristaltic pumps to achieve the desired organic loading rate and HRT. Once the reactors reached the steady state, H_2_ gas was supplemented to each reactor with two stainless steel diffusers (pore size 2 μm) at the rate of 1 mL/min. To ensure efficient H_2_ utilization, the gas phase of the reactors was constantly recirculated into liquid phase with peristaltic pumps. Throughout the experiment, biogas production was recorded with water-replacement gas metres; biogas composition was measured using a gas chromatograph (Mikrolab, Aarhus A/S, Denmark), equipped with a thermal conductivity detector (TCD). The volatile fatty acids and ethanol were measured with a gas chromatograph (Shimadzu GC-2010 AF, Kyoto, Japan), equipped with a flame ionization detector (FID) [19]. Biomass formation was estimated through volatile suspended solids measured according to the Standard Methods for the Examination of Water and Wastewater [20]. All determinations and measurements were done in triplicate samples.

### Sample collection and sequencing

The liquid samples were acquired from the triplicate reactors before, 18 hours after and 36 days after H_2_ addition (Sample point 1, 2, and 3, respectively). For all the samples, the genomic DNA was extracted with PowerSoil® DNA Isolation Kit and the total RNA was extracted PowerMicrobiome® RNA Isolation Kit (Mo Bio Laboratories, Inc., Carlsbad, USA). All the extractions were performed with additional phenol cleaning steps in order to improve the quality of the extractives. The ribosome RNA was removed from total RNA samples with Ribo-Zero® rRNA Removal Kit (Bacteria) (Illumina, San Diego, USA). The DNA and RNA samples were sent to Ramaciotti Centre for Genomics (UNSW, Sydney, Australia) for cDNA construction, library preparation, and sequencing (Illumina NextSeq).

### Genome-centric metagenomics

The DNA sequences from 9 samples were filtered with Trimmomatic software [21], co-assembled with metaSPAdes [22], and atomically binned with MetaBAT [23]. The quality of the metagenome-assembled genomes (MAGs) were examined with CheckM [24] and evaluated with a MAG quality standard developed by Genomic Standards Consortium [25]. The average nucleotide identity analysis (ANI) was performed against all the genomes that were deposited in NCBI Reference Sequence Database [26]. The genomes hits with ANI higher than 97% were used to classify the MAGs at the species level [27, 28]. The putative taxonomy classification of the unclassified MAGs were further assessed based on ubiquitous proteins with PhyloPhlAn [29]. The genes of the co-assembled metagenome were predicted and annotated with Integrated Microbial Genomes & Microbiomes (IMG) [30]. For more comprehensive methanogenic pathway reconstruction, all archaeal MAGs were resubmitted to IMG as genomes assembled from metagenome for gene prediction and annotation.

The microbial communities were profiled through reads recruitment from the sequencing samples. The average coverage of MAGs in each metagenome sample was calculated based on the number of reads aligned by Bowtie2 [31] and the detailed procedure were described by Campanaro et al. (2016) [32]. The relative abundance of the MAGs in a community was determined with the average coverage of the MAG in one metagenome sequencing sample, according to:

$$ Relative\kern0.17em abundance\;{(MAG)}_{sample1}=\frac{average\kern0.17em coverage\;(MAG)}{\varSigma_{All\; MAGs\kern0.17em in\kern0.17em sample1} average\kern0.17em coverage} $$

### Genome-dissected metatranscriptomics

The sequenced transcripts were aligned to assembled metagenomes with Bowtie2 and quantified with HTSeq-count [31, 33]. Therefore, instead of de-novo assembling the RNA sequences, the metatranscriptomes inherited the annotation from the corresponding metagenomes. The expression level of genes was evaluated by reads per reads per kilobase of exon model per million mapped reads (RPKM) [34].For comparison purpose, the RPKM numbers were normalized considering the expression level of methyl-coenzyme M reductase gene (subunit alpha) and CH_4_ production rates of the reactors during the time that each sample was collected. Moreover, the metatranscriptomes were dissected according to the binning results in order to generate individual expression profiles for MAGs. The overall activity of a MAG was evaluated by the average gene RPKM within the genome. The relative activity of MAGs in a community was measured according to a similar formula as relative abundance:

$$ Relative\kern0.17em abundance\;{(MAG)}_{sample1}=\frac{average\kern0.17em gene\kern0.17em RPKM\;(MAG)}{\varSigma_{All\; MAGs\kern0.17em in\kern0.17em sample1} average\kern0.17em gene\kern0.17em RPKM} $$

The comparison between the relative abundance and activity of a MAG suggested its activity level. More specifically, a low abundance/ activity ratio represented an active member, who undertook many microbial metabolisms with few numbers of cells. The overall microbial community composition was visualized by Anvi’o [35].

The gene expression profiles derived from genome-dissected metatranscriptome were also used to indicate the functional role of each MAG during the methanogenic process. Genes were categorized based on KEGG modules and the average RPKM of all the genes was calculated for each module. The differential expression of each gene in coordination to the H_2_ addition was examined using edgeR package [36]. Other statistical tests (Student’s t-tests and correlation tests) were performed with Excel.

Specific metabolisms, i.e. methanogenesis and acetate uptake, were tentatively distributed to individual MAGs based on the expression level of signature genes. For instance, the methanogenic activity of individual archaeal MAGs was determined based on the expression level of MAG-specific *mcrA* comparing to the overall expression of all *mcr*A [37]*.* Moreover, MAG-specific acetate kinase (*ack*) as well as acyl-CoA synthetase (*acs*) were used to correlate the acetate metabolism to individual members among the microbial community.

### Data availability

The raw sequence data were deposited on sequence read archive with accession no PRJNA525781, The biosample metadata were deposited in Genomes OnLine Database (GOLD) as study Gs0128993.The metagenome annotation was deposited as analysis project Ga0214976. The annotation of methanogen MAGs was deposited as analysis projects Ga0214977, Ga0214981, Ga0214989, Ga0214990 and Ga0214991.

## Results

### Methanogenic microcosms enriched by acetate and H_2_

**Fig. 1:**
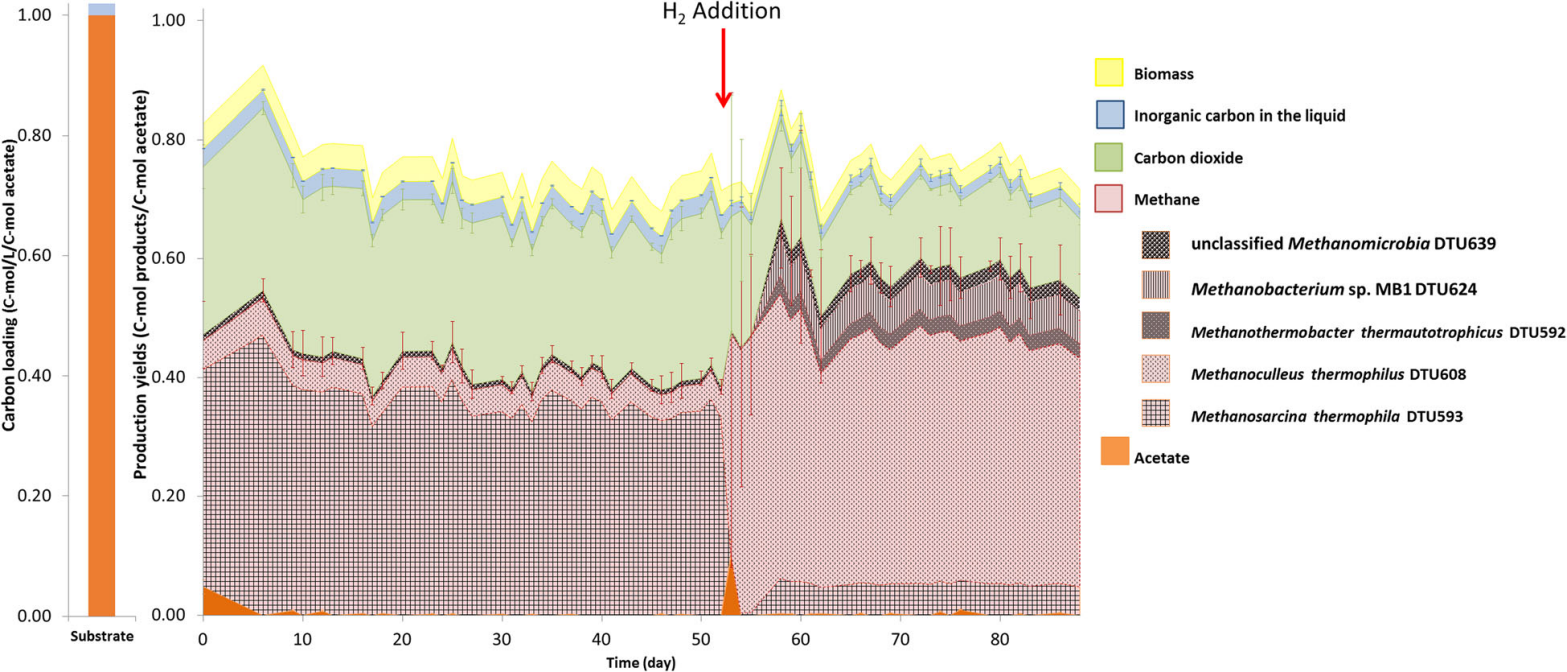
Digestion profile before and after H_2_ addition in the reactors. The presented values and standard deviations are calculated from three reactors as biological triplicates. The single bar graph on the left represents the carbon source provided to the system. The CH_4_ production activity was allocated into five archaeal metagenome-assembled genomes (MAGs) based on the expression level of MAG-specific methyl coenzyme M reductase gene (*mcrA,* alpha unit)

The tractable low-complexity methanogenic microbial communities were obtained from triplicate lab-scale continuous biogas reactors operated under thermophilic conditions providing acetate as the only organic carbon source. After establishment of stable conditions, external H_2_ gas was injected into all reactors with stainless steel diffusers to assess the microbes’ affinity to H_2_ partial pressure. During the entire experimental operation, the pH in each reactor was self-stabilized within the optimal range of methanogenesis (7-7.5). The triplicate reactors performed consistently during the two steady operational conditions (Sample Points 1 and 3). Nevertheless, a significant discrepancy among triplicate reactors was observed during the transition before and after H_2_ addition (Sample Point 2), which was mainly attributed to the instability of the microbial community adaptation process. CH_4_, along with inorganic carbon compounds including CO_2_, bicarbonate (HCO_3_^-^) and carbonate (CO_3_^2-^), were the main digestion products. The digestion profiles are described as mol of carbon contained in each products (Fig. 1). In addition, approximately 4% of carbon (mol of carbon in biomass / mol as carbon in acetate) was used to build microbial biomass. The methanation process was extremely efficient as, less than 0.5% of the carbon (mol of carbon in acetate / mol as carbon in acetate) left the system as undigested acetate. All of the injected H_2_ was consumed and the CH_4_ yield significantly increased from 299.8±4.4 mL/g acetate to 409.3±14.6 mL/g acetate (Fig. 1, Additional file 1).

### Metagenome-assembled genome reconstruction and taxonomy assignment

**Fig. 2:**
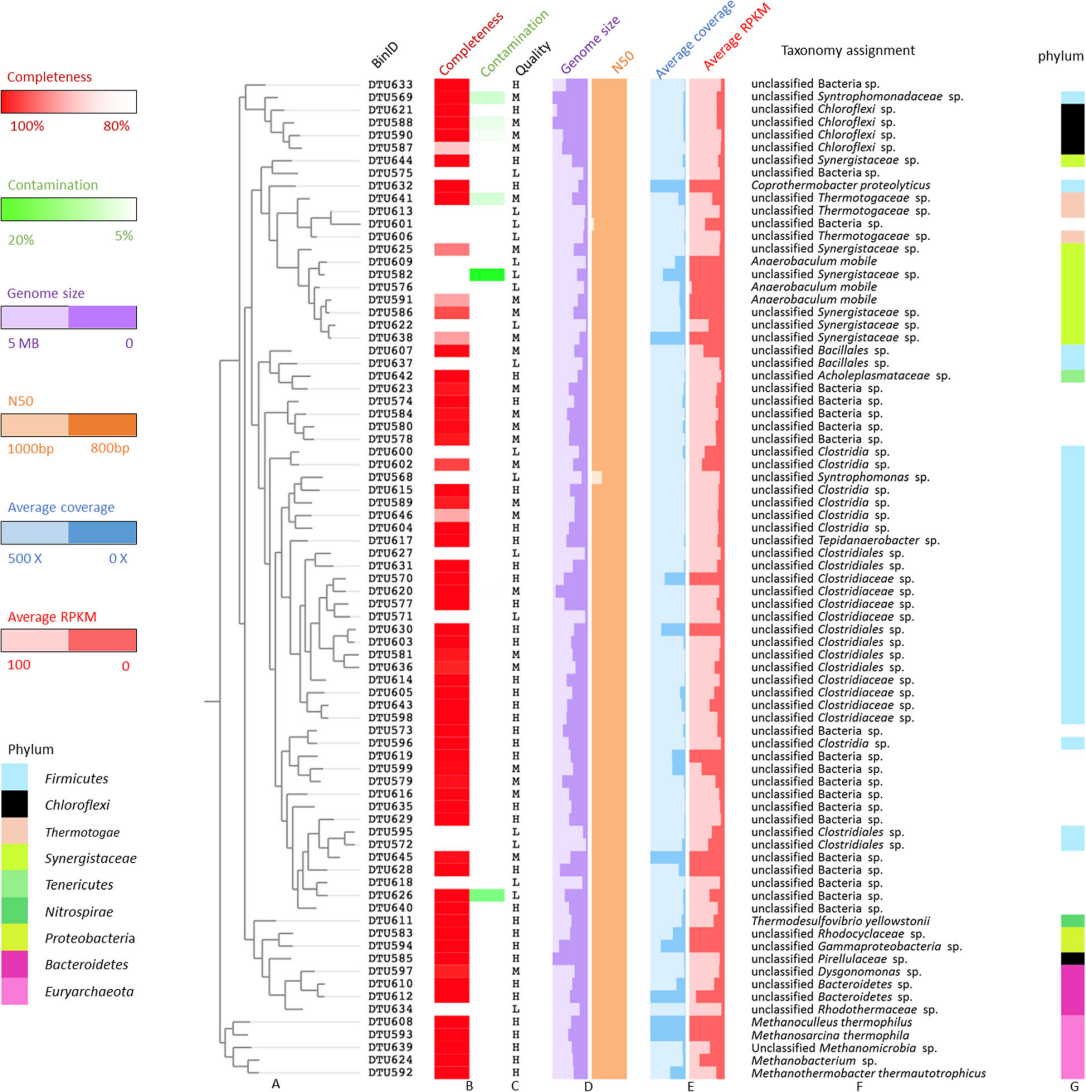
Metagenome assembled genomes (MAGs) and their phylogeny relationships. (**a**) Phylogenetic tree of 79 MAGs inferred from the phylogenetic signal extracted from 400 taxonomic informative proteins. (**b**) The completeness and contamination values assessed by CheckM determined using genes that are ubiquitous and single-copy within a phylogenetic lineage. (**c**) The genome quality was used to classify MAGs according to indications provided by the Genomic Standards Consortium. “H” represents high quality, “M” represents medium quality, and “L” represents low quality. (**d**) The genome size and N50 value determined considering the contigs assigned to the MAGs. (**e**) The average coverage and RPKM of each MAG in 9 samples. (**f**) Taxonomy classification assigned with ANI calculation determined against genomes of microbial isolates deposited at the NCBI database, as well as putative classification according to the phylogenetic signal extracted from 400 taxonomic informative proteins. (**g**) Taxonomy classification at the phylum level

Samples for shotgun sequencing were collected from triplicate reactors at three time points:1) before H_2_ addition, 2) 18 hours after H_2_ addition, and 3) 36 days after H_2_ addition. Point 1 and Point 3 were chosen during the operational steady states where the CH_4_ production rate of each reactor varied less than 10% for 10 consecutive days. Sequences from all samples were co-assembled and automatically binned in order to extract metagenome assembled genomes (MAGs). In total, 79 MAGs were extracted with total coverage ranging from 5× to 9595× (Fig. 2, Additional File 1). According to the completeness and contamination values assessed by CheckM, and the quality standards developed by Genomic Standards Consortium [25], 36 MAGs were assigned to the “high-quality” group, 25 MAGs to the “medium quality” group, and 18 MAGs to the “low quality” group. It is noteworthy that over 95% of total shotgun sequences including DNA and RNA in all samples, could be aligned to the medium-high quality MAGs, suggesting that the majority of the microbial diversity has been recovered with the assembly and binning process.

Five nearly complete archaeal MAGs were present in the community (> 98% complete) , four out of which were characterized at species level as *Methanothermobacter thermautotrophicus* [38, 39] DTU592, *Methanosarcina thermophila* [40] DTU593, *Methanoculleus thermophilus* [41] DTU608 and *Methanobacterium* sp. MB 1[42] DTU624 (Additional File 2). The unknown archaeal MAG (unclassified *Methanomicrobia* DTU639), which was also previously found in biogas reactors [32], could be assigned to a member of class *Methanomicrobia* based on tentative phylogenetic classification. In contrast to archaeal MAGs, 51 out of 56 bacterial MAGs could only be classified at the family or higher taxonomic level. According to the relative abundance, more than 90% of the microbial community could be represented by 18 most abundant MAGs, 15 of which belonged to domain *Bacteria*. Among bacteria, 11 MAGs were assigned to *Firmicutes* (4 MAGs)*, Bacteroidetes* (2 MAGs) *Synergistetes* (2 MAGs), *Proteobacteria* (2 MAGs), and *Thermotogae* (1 MAG). The remaining four MAGs were unclassified Bacteria spp. (Additional File 3).

### Microbial community composition and transcriptional activity

**Fig. 3:**
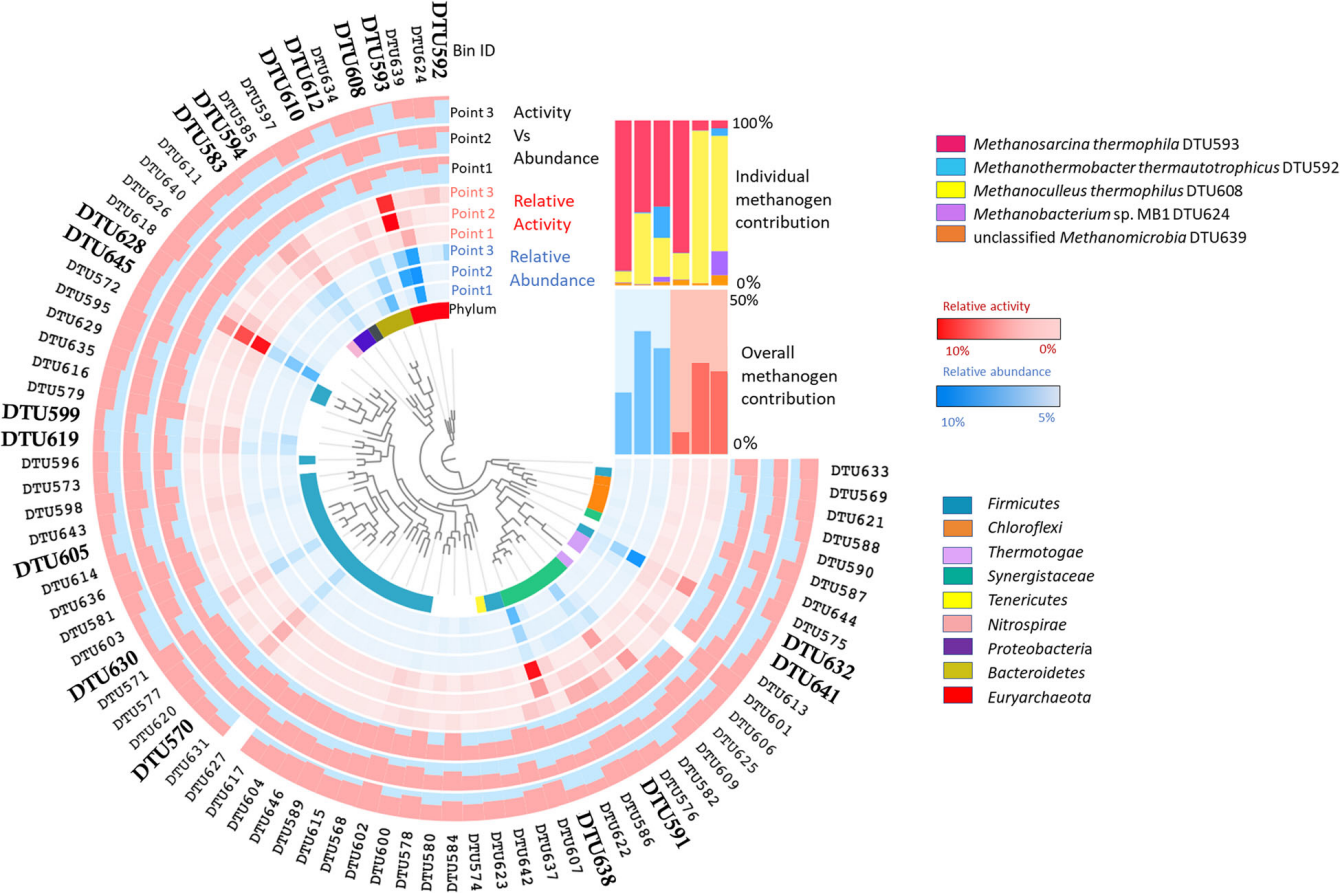
Microbial community composition and transcriptional activity profiles. MAGs present in top deciles are now highlighted.The inner blue circles (before, shortly after, and long after H_2_ addition) represent relative abundances of each MAG calculated from the average coverage in sequenced samples. The middle red circles (before, shortly after, and long after H_2_ addition) represent relative activities of each MAG calculated from average gene expression levels (RPKM). Comparisons between relative abundance and activity are represented in the three outer circles

The microbial community composition and the transcriptional activity profiles were determined using the average genome coverage of each MAG and the average gene expression level (reads per kb per million mapped reads, RPKM) of all protein-coding genes in each MAG (Fig. 3, Additional File 4 and 5 ). Interestingly, a robust bacterial activity was observed in the reactor, although the present acetoclastic methanogens *(M. thermophila* DTU593) could theoretically undertake the majority of the acetate methanation process. In fact, the five methanogens constituted only a small part of the total microbial community, which is 19–37% of the abundance and 7–27% of the activity. It was surprising that methanogens constituted the minority both in respect to relative abundance and activity since only methanogenic substrates (acetate and H_2_-CO_2_) were fuelling the process. Before H_2_ addition, the most abundant MAG (DTU593) among the entire microbial community was classified as *Methanosarcina thermophila,* accounting for 17% of the total community (Additional File 4). However, its activity was calculated as 5.3% among the entire microbial community (Additional File 5). The relatively high RNA/DNA ratio indicated a high cellular protein synthesis potential of *M. thermophila* DTU593 [43], suggesting a possible high growth rate under this condition [44, 45]. In contrast, unclassified Bacteria sp. (DTU645) and unclassified *Synergistaceae* sp*.* (DTU638), which were the second and third most abundant MAGs (accounting for 12% of the community each), were responsible for 19% and 18% of the activity respectively (Additional File 4 and 5). After H_2_ addition, the microbial abundance (based on genome coverage) and transcriptional activity (based on average gene RPKM) profiles changed significantly as a result of community adaptation .The overall archaeal activity changes correlated with the CH_4_ production rate of the reactor in steady state (*R*^2^=0.84), whereas the correlation between the overall archaeal relative abundance and steady state CH_4_ production was lower (*R*^2^=0.53) (Additional File 5). In addition to methanogenic archaea, the supplemented H_2_ also reshaped the bacterial community. The most significant change was the increase of *Coprothermobacter proteolyticus* DTU632, which became the most abundant MAG, accounting for 19% of the total community. Interestingly, *C. proteolyticus* DTU632 only contributed to 6.8% of activity, which was lower than the hydrogenotrophic methanogen *M. thermophilus* DTU608 (18.6%) and unclassified Bacteria sp. DTU645 (7.2%) (Additional files 4 and 5).

### Metabolism of the methanogens

Acetate, the only organic carbon source supplied to the reactors, was taken up by the microbes through two pathways: inversed phosphotransacetylase-acetate kinase pathway (PTA-ACKA) and AMP-forming acyl-CoA synthetase pathway (AMP-ACS). Thus, acetate utilization by individual microbes could be estimated according to the expression level of MAG-specific acyl-CoA synthetase (*acs*) and acetate kinase (*ack*) genes (Additional File 7). The main archaeal acetate consumer was *M. thermophila* DTU593, which consumed less than 50% of the acetate supplied to the reactors through inversed PTA-ACKA before H_2_ addition. After H_2_ addition, the expression level of *M. thermophila-*specific *ack* was decreased significantly, while expression of bacterial *ack* was increased. Considering that CH_4_ was produced by the five archaea, the methanogenic activity was tentatively distributed among them (expressed as %) based on the expression level of MAG-specific *mcrA* (Fig. 2, Additional File 8). Before H_2_ addition, the methanogenic activity was highest in *M. thermophila* DTU593 (86%) and *M. thermophilus* DTU608 (11%). After reaching the steady state, external H_2_ gas was supplied in order to trigger a metabolic shift towards hydrogenotrophic methanogenesis. The amount of H_2_ injected into the reactor was chosen stoichiometrically to reduce half of the CO_2_ that was produced from acetate during the methanogenic process. The addition of external H_2_ gas changed the methanogenic activity of archaeal MAGs. Specifically, the activity of *M. thermophilus* DTU608 was significantly enhanced and it became the main methanogenic contributor in the microbial community shortly after the H_2_ addition (98% of methanogenic activity). By the end of the experiment, the reactor stabilized in a new operational steady state, during which 56 ± 0.4% of acetate-carbon was converted to CH_4_ (16% more compared with previous states). After long-term adaptation to the H_2_ addition, although *M. thermophilus* DTU608 maintained its dominance (71%), a small but significant fraction of methanogenic activity was taken over by *M. thermophila* DTU593 (9%) and other hydrogenotrophic methanogens (15%).

**Fig. 4:**
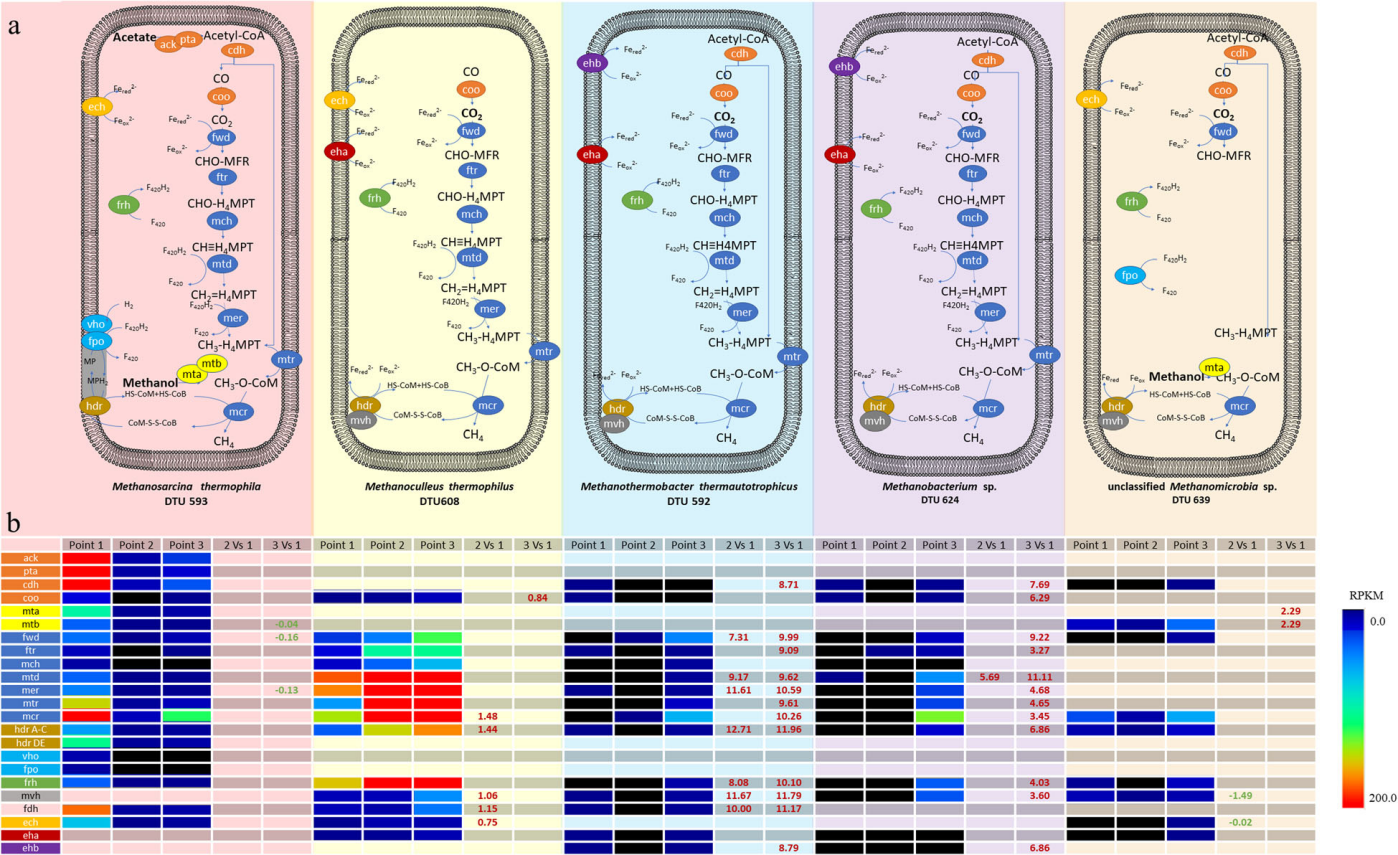
**a** Methanogenic pathway reconstructions in five archaeal MAGs. CoA, coenzymeA; MFR, methanofuran; H_4_MPT, tetrahydrosarcinapterin; HS-CoM, coenzyme M; HS-CoB, coenzyme B; MP, methanophenazine; Fe_ox,_ Ferredoxin; F_420_, coenzyme 420; *ack*, acetate kinase; *pta*, phosphate acetyltransferase; *cdh*, acetyl-CoA decarbonylase; *coo*, carbon-monoxide dehydrogenase; *mta*, methano-specific coenzyme M methyltransferase; *mtb*, methylamine-specific coenzyme M methyltransferase; *fwd*, formylmethanofuran dehydrogenase; *ftr*, formylmethanofuran--tetrahydromethanopterin N-formyltransferase; *mch*, methenyltetrahydromethanopterin cyclohydrolase; mtd, methylene tetrahydromethanopterin reductase; *mer*, F_420_-dependent methylenetetrahydromethanopterin dehydrogenase; *hdr* A-C, heterodisulfide reductase subunits A-C; *hdr* DE, heterodisulfide reductase D and E; *vho*, methanophenazine-reducing hydrogenase; *fpo*, F_420_H_2_ dehydrogenase; *frh*, coenzyme F_420_ hydrogenase subunit; *mvh*, F_420_-non-reducing hydrogenase; *fdh*, formate dehydrogenase; *ech*, *Escherichia coli* hydrogenase 3; *eha*, energy-converting hydrogenase A; *ehb*, energy-converting hydrogenase B. **b** The expression of genes related to methanogenesis. The colors represent different steps of methanogenic pathways. Significant up (red) and down (green) regulation of genes (evaluated with edgeR) is indicated by colored numbers

Pathways related to methanogenesis and relevant energy conservation systems were reconstructed in all archaeal MAGs (Fig. 4). The expression levels of those genes (normalized according to the expression level of *mcrA* gene and CH_4_ production rate) were examined before, shortly after and long after H_2_ addition (Additional File 8). *M. thermophila* DTU593 expressed all the genes involved in hydrogenotrophic, methylotrophic and acetoclastic methanogenesis, indicating its multi-trophic functional role in anaerobic digestion. In particular, the expression of methylamine/methanol-specific coenzyme M methyltransferase genes (*mta*, *mtb*) suggested a considerable contribution of methylotrophic pathways (Additional File 8). For energy conservation, *M. thermophila* DTU593 obtained the electron from intermediate H_2_ through methanophenazine-reducing hydrogenase (*Vho*), coenzyme F_420_-reducing hydrogenase (*Frh*), and *Escherichia coli* hydrogenase 3 (*Ech*). The electrons provided by *Ech* were transferred to ferredoxin and used for CO_2_ reduction. The electrons carried by F_420_H_2_ were not only used for CHO-H_4_MPT reduction in hydrogenotrophic methanogenesis but also transferred to methanophenazine through F_420_H_2_ dehydrogenase (*Fpo*). Finally, methanophenazine reduced by *Fpo* and *Vho* transferred the electrons to CoM-S-S-CoB through the membrane-bound heterodisulfide reductase (*hdr* DE).

In contrast, the methanogenic activity of *M. thermophilus* DTU608 was restricted to hydrogenotrophic pathways (Fig. 4). *M. thermophilus* DTU608 lacked cytochromes but possessed the electron bifurcation system, which allows coupling CO_2_ reduction and CoM-S-S-CoB reduction with *Mvh*-*Hdr* complex oxidation. In addition, *M. thermautotrophicus* DTU592 and *Methanobacterium* sp. gradually increased their relative abundance and activity only after long term operation. These two ‘slowly emerged’ archaeal MAGs encoded core hydrogenotrophic methanogenesis pathways similar to *M. thermophilus* DTU608*;* however, they possessed different hydrogenase complexes for H_2_ uptake. An important difference is that both *M. thermautotrophicus* DTU592 and *Methanobacterium* sp. DTU624 used energy converting hydrogenase B (*Ehb*) instead of *Ech*, which was present in *M. thermophilus* DTU608 (Fig. 4, Additional file 9).

*Ehb* was proven to be related to autotrophic CO_2_ assimilation, which could confer an advantage to *Methanobacteriaceae* spp. by increasing its relative abundance in the microbial community during long-term organic carbon starvation [46]. Moreover, both *M. thermautotrophicus* DTU592 and *Methanobacterium* sp. DTU624 significantly upregulated carbon monoxide dehydrogenase (*coo*) and acetyl-CoA decarbonylase/synthase (*Cdh*) genes, supporting carbon fixation activity for autotrophic growth (Fig. 4, Additional File 8), while *M. thermophilus* DTU608 relied on external acetate (heterotrophic) as indicated by the expression of NDP forming acyl-CoA synthetase genes.

### Metabolism of the bacteria

**Fig. 5:**
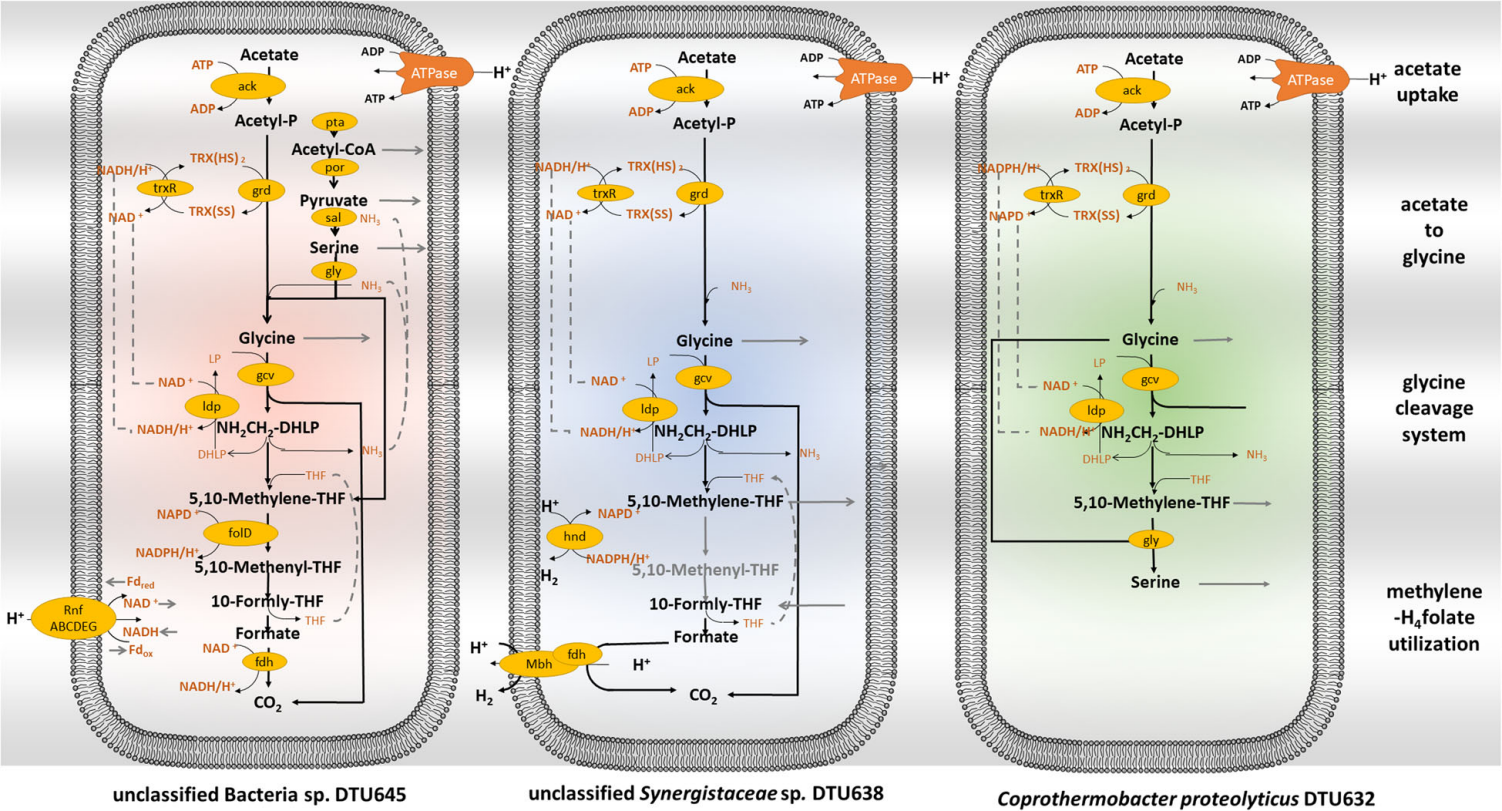
Acetate utilization pathways in the three most abundant bacterial MAGs. Black arrows represent the reactions mediated by highly expressed genes. Grey arrows represent the metabolites flowing to other metabolic pathways in the cell. Black solid lines represent the reaction mediated by actively expressed genes in the MAG. Grey solid lines represent the metabolites flowing to other metabolic pathways in the cell. Grey dashed arrow lines connect the same compounds/cofactors, which are recirculating in the cell

More than 50% of the acetate, which was not taken up by archaea, was metabolized by bacterial members in the community (Additional File 7). According to the transcriptional activity of formyltetrahydrofolate synthetase gene (*fhs*), about half of the bacteria community (31 out of 79) were indicated to have a syntrophic lifestyle in association with methanogenic Archaea [47, 48]. Interestingly, the genes encoding the enzyme to directly break the bonds between the carbonyl and methyl branch in the acetate (acetyl-CoA decarbonylase , *cdh*) were expressed at an extremely low level (not significant according to edgeR normalization) in all bacterial MAGs (Additional File 10). This result indicated that these bacteria may have acetate utilization pathways other than the conventional Wood-Ljungdahl (WL) pathway, similar to the alternative pathway previously proposed in *Thermotogae* spp. [49]. High expression levels of *ack* and glycine decarboxylase genes (*gcvP*) were found in MAGs belonging to diverse taxa, indicating that the glycine cleavage system proposed for the *Thermotogae* phylum might be more widely used for bacterial acetate utilization (Additional File 10). Bacteria adopted versatile strategies to transform acetate to glycine, to further metabolize the intermediates released from the glycine cleavage system, as well as to conserve energy. The detailed acetate degradation pathways were proposed based on highly expressed genes (50% quantile among all the genes expressed in the genome) in the most abundant acetate consuming bacterial MAGs (Fig. 5, Additional File 11, 12 and 13).

The *Thermotogae*-like acetate utilization pathway was reconstructed in unclassified Bacteria sp. DTU645, which phylogenetically clustered with *Firmicutes* (Fig. 1). The high expression level of glycine reductase gene (*grd*) in unclassified Bacteria sp. DTU645 suggested an alternative path for acetate to enter the glycine cleavage system, where acetyl phosphate was directly converted to glycine through reversed glycine reduction (Additional File 11). The glycine reductase gene was found highly expressed in many other bacterial acetate utilizers, e.g. unclassified *Synergistaceae* sp. DTU638 and *C. proteolyticus* DTU632 (Additional File 12 and 13). The glycine cleavage system catalysed the decarboxylation of glycine and released methylene-tetrahydrofolate, NH_3_ and CO_2_. The methylene-tetrahydrofolate could be further oxidized through a partial reversed WL pathway in unclassified *Synergistaceae* sp. DTU638 and unclassified Bacteria sp. DTU645, having CO2 as the final product (Additional File 11 and 12). Interestingly, the gene set mediating methylene-tetrahydrofolate oxidation was completely absent in *C. proteolyticus* DTU632, whose genome was 100% complete according to CheckM (Additional File 2). *C. proteolyticus* was previously characterized as a proteolytic bacterium that produces acetate, CO_2_ and H_2_ as main fermentative products [48]*.* However, its high relative abundance and activity in this study indicated its involvement in the acetate metabolism with additional H_2_ supplements. Considering *C. proteolyticus* is known to metabolize amino acids, a Stickland-like reaction was tentatively reconstructed in strain DTU632 after considering the highly expressed genes (Fig. 5, Additional file 13). Specifically, the methylene-tetrahydrofolate released from the glycine cleavage system was combined with another glycine to create serine, and eventually entered the pathways for amino acid metabolism. In all the proposed pathways, the electrons were balanced from acetate-uptake to glycine decarboxylation, and additional electron disposal was required for further oxidation of methylene-tetrahydrofolate. For unclassified *Synergistaceae* sp*.* DTU638, the electron was disposed of as H_2_ as suggested by the high expression of membrane-bound hydrogenase and Fe-S-cluster-containing hydrogenase (Additional File 12), which explained why H_2_ inhibited the activity of unclassified *Synergistaceae* sp*.* DTU638. As a consequence of external H_2_ addition, the acetate uptake activity was taken over by unclassified Bacteria spp. Unlike unclassified *Synergistaceae* sp*.* DTU638, the formate dehydrogenase operon of unclassified Bacteria sp. DTU645 did not contain a hydrogenase gene (Additional File 11), suggesting the electrons could be disposed of in other forms than H_2_. This observation explained the increment in relative abundance of DTU645 and other bacterial members after H_2_ addition.

### Overall metabolism of microbial community

In order to maintain the methanogenic activity of the microbial community, a syntrophic behaviour is needed to synthesize numerous metabolites. The holistic microbial community activity could be evaluated by the average RPKM of genes in each KEGG module. An overall shift of the microbial activity was observed in the majority of the KEGG modules after H_2_ addition. Specifically, the expression level of the KEGG modules related to methanogenesis, including both reactions directly linked to CH_4_ formation and biosynthesis of cofactors (F_420_) increased roughly 1.5-fold after H_2_ addition (Additional File 14). Moreover, H_2_ also enhanced the activity of the glyoxylate cycle and the biosynthesis of lipids and specific amino acids (Additional File 14).

Although both abundance and activity of individual MAGs changed significantly in the different H_2_ adaptation stages, ubiquitous metabolic pathways were found to be essential for maintaining the complex microbial community. Specifically, the results showed that the core metabolisms carried out by the dominant bacteria community before H_2_ addition could be maintained by other members proliferating after H_2_ addition (Additional File 14). These metabolic steps were catalysed by proteins encoded by constitutively expressed genes that maintain basic cellular function, such as biosynthesis, energy conservation, repair, and regulatory systems.

**Fig. 6:**
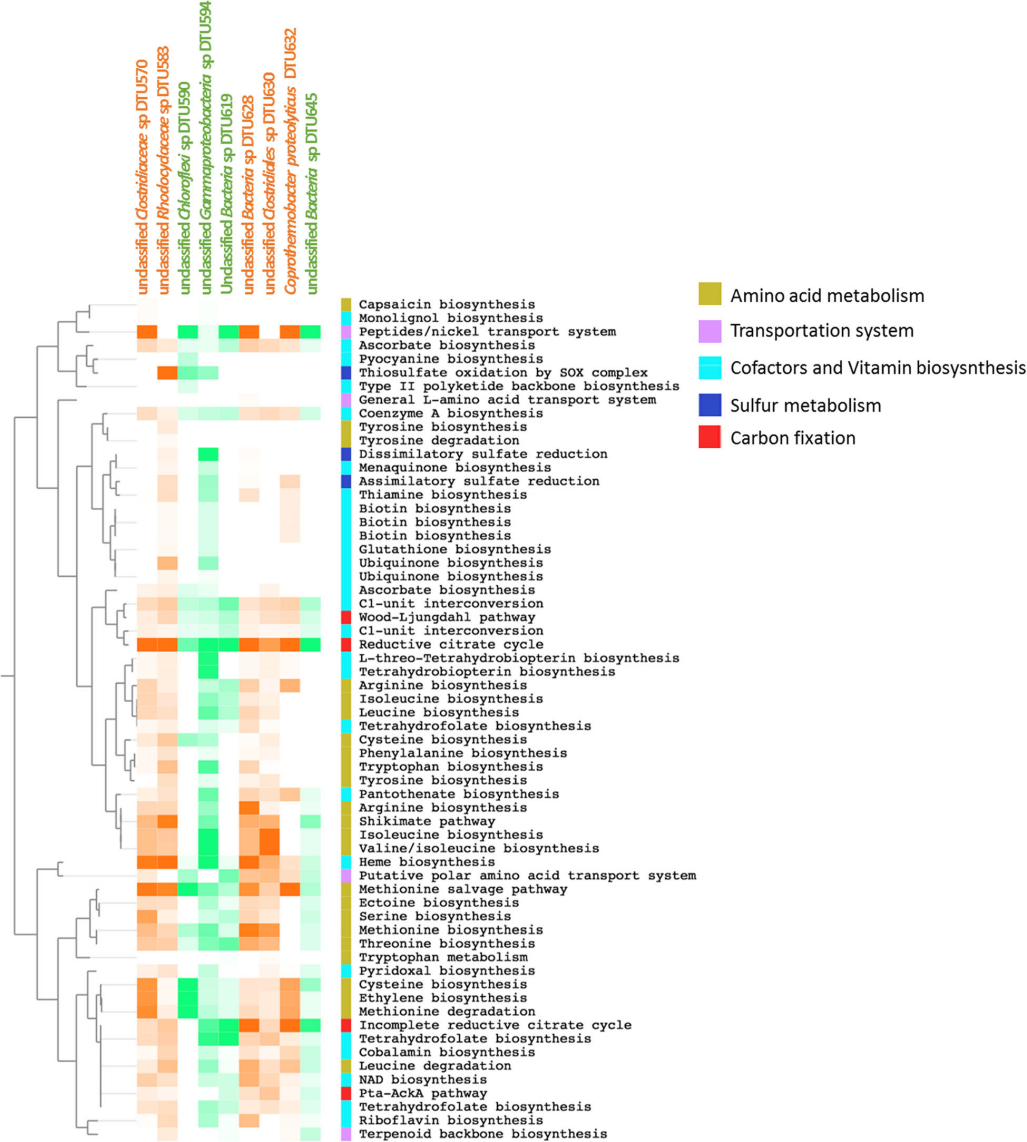
Expression profiles (normalized by coverage) of most abundant bacterial MAGs and relevant KEGG modules. MAGs increasing in relative abundance after H_2_ addition are indicated in orange. MAGs decreasing in relative abundance after H_2_ addition are indicated in green. The metabolic categories are indicated with colours

The investigation of each individual MAGs’ expression profile showed that the biosynthesis of common cofactors such as coenzyme A, NAD and riboflavin were evenly expressed in the dominant microbes, whereas the biosynthesis of several energy-expensive amino acids [50] and cofactors (such as biotin) were absent from some MAGs (Fig. 6). Specific metabolic traits are suggested for individual microbes based on their gene expression profile. For example, *C. proteolyticus* DTU632 lacked efficient pathways for electron disposal and energy-efficient acetate catabolism but showed high activity of reductive citric acid cycle. Therefore, *C. proteolyticus* might grow as energy-expensive amino acid auxotrophs to reduce the biosynthetic burden. The high expression of many amino acid transport systems indicated that the growth of *C. proteolyticus* DTU632 was supported by external amino acid uptake, such as tryptophan, tyrosine, and cysteine, (Additional File 13). The expression profiles of unclassified Bacteria sp. DTU628, unclassified *Clostridiales* sp. DTU630, and unclassified *Rhodocyclaceae* sp. DTU583 implied that these bacteria could synthesize relevant amino acids during H2 addition (Fig. 6). Another interesting observation was that the genes involved in the biosynthesis of biotin were only found in unclassified *Clostridiaceae* sp. DTU570, unclassified *Gammaproteobacteria* sp. DTU594, and unclassified *Clostridiales* sp. DTU630. It was previously demonstrated that the growth of some methanogens required an external supply of biotin [51]. Considering the consistent expression of genes encoding biotin-specific transporters in the methanogens, biotin auxotrophy might have forced methanogens to scavenge metabolic products for methanogenesis, thereby leading to syntrophic behaviour between bacteria and archaea.

## Discussion

The combination of genome-centric metagenomics and metatranscriptomics successfully revealed individual functional roles of microbial members in methanogenic microcosms. The results assigned a multi-trophic role to *Methanosarcina thermophila*, suggesting its ability to perform simultaneous methanogenesis from acetate, CO_2_ and methanol/ methylamine. Although the use of cytochromes in *M. thermophila* would impose thermodynamic limitations to compete for H_2_ during low H_2_ partial pressure [52], *Fpo-Hdr* mediated heterodisulfide reduction promoted the activity of *Frh*, leading to the activation of the hydrogenotrophic pathway. Therefore, the H_2_ produced as an intermediate during anaerobic digestion not only promote the growth of hydrogenotrophic methanogens but also provide a favourable ecological niche for *M. thermophila.* The complex association between acetoclastic methanogens and acetate-oxidising bacteria could be one cause of functional redundancy in microbial communities involved in biomethanation. In this experiment, although *M. thermophila* had the metabolic potential to perform methanogenesis through a mainly acetoclastic pathway, a bacteria-dominated microbial consortium was formed, which resulted in a multi-trophic methanogenesis strategy. The results also underlined the importance of methanol/methylamine methanogenic pathways, although significant methanol concentrations were not detected during the process. In fact, the methanol/ methylamine-specific methanogens were previously identified as pivotal members in many other biogas reactors fed with manure [32]. From this result, we believe that the maintenance of the relevant metabolites (such as methanol/methylamine) at low concentration in an efficient anaerobic digestion system. The addition of external H_2_ greatly enhanced the activity and the relative abundance of hydrogenotrophic methanogens, including *M. thermophilus*, whose activity was inherent in the microbial community before H_2_ addition and *Methanobacteriaceae* spp, which was nonexistent before H_2_ addition but significantly increased later after a long period of adaptation to external H_2_. The stimulation of *Methanomicrobia* members was in accordance with previous research, where anaerobic digestion systems were exposed to different H_2_ partial pressures [53–55]. For instance, a study on biogas biological upgrading systems [54] concluded that the microbial community would turn over from a “*Methanoculleus-*dominated” microbial community to a “*Methanothermobacter*-dominated” community after a 2-year stable operation with external supplemented H_2_. Several hypotheses were proposed to explain the methanogens differing affinities to H_2_ concentration; these hypotheses were based on gene expression regulation, or considered energy conservation strategies and syntrophic associations with bacterial partners [12]. This work suggests that the competition among the different hydrogenotrophic methanogens can be explained by a bargain between methanogenic activity and autotrophic growth. The hydrogenase genes encoded by *Methanobacteriaceae* spp. (*ehb*) might especially support growth with external H_2_ and promote growth during long-term H_2_ adaptation and limited carbon sources.

The bacterial metabolic pathways were essential for their contribution to acetate oxidation, as well as for their role in maintaining the microbial community structure. Bacterial acetate oxidation under anaerobic conditions is postulated to be performed through the reductive WL pathway, which was annotated in the known syntrophic oxidizer, *Syntrophaceticus schinkii* [56]*.* However, it was also recently found that many genomes from acetate utilizers, including both MAGs and isolates, possessed only a subset of WL pathway genes [49, 57]. The results of this study showed extensive use of a glycine cleavage system by many members of the community to circumvent the direct break of the carbonyl and methyl carbon bonds of acetate. The glycine cleavage system could be used in the previously proposed manner, where it was combined with a partial WL pathway to produce CO_2_/H_2_ and support the syntrophic activity with hydrogenotrophic methanogens [49]. Moreover, in the present study, a completely new Stickland-like path was proposed for *C. proteolyticus* DTU632. Unlike the conventional Stickland reaction where the amino acids were provided to the microbes as a carbon source, in this newly proposed pathway, acetate was converted to glycine which served as both an electron donor and acceptor for further metabolism. The oxidation of carbonyl groups was performed through the glycine cleavage system and the methyl carbons were used for amino acids biosynthesis as previously suggested in organohalide-respiring *Dehalococcoides mccartyi* [58]*. C. proteolyticus*’s capability to utilize acetate was not revealed in studies performed on pure cultures [59] and this metabolic trait might only be activated under specific conditions. The current experiment imposed a selective pressure on *C. proteolyticus*, where acetate was the sole organic source, external H_2_ was supplemented, and microbial partners were present to form syntrophic relationships. This finding encourages future studies to explore metabolic potential in diverse environments and to prove that the functional roles of individual members of a microbial community could go beyond the physiological characterization of the corresponding isolates. Lastly, some crucial transcriptomic activities, such as biosynthesize of amino acids and cofactors, were absent in the most abundant MAGs, which indicated potential exchange of carbon sources, amino acids, and cofactors among bacterial and archaeal members. These results underlined the importance of auxotrophy in the microbial communities, which was previously proposed to reduce biosynthetic burden [60, 61]. This finding may be considered one of the most important reasons for maintaining/forming a complex microbial community even during growth on simple substrates (e.g. acetate). Auxotrophy could also provide explanations for previous observations, as for example, the unexpected proteolytic activity of *C. proteolyticus* [15], which was observed during cellulose degradation (without protein as substrate), and required an external source of energy-expensive amino acids.

## Conclusions

The combined genome-centric metagenomics and metatranscriptomics strategy used in the present work was extremely informative to characterize unknown microbial communities and elucidate the metabolic activity of individual microbial species. Especially, the distribution of metabolic activities based on genome-dissected metatranscriptomes directly revealed the contribution of individual MAGs to the global activity of the microbiome. The novel microbial insights illustrated in the current study expanded the current knowledge regarding metabolisms in methanogenic systems and the results obtained can open new horizons for future microbial ecology studies of interspecies competition or symbiosis.

## Supplementary information

**Additional file 1.** The genome quality of all MAGs.

**Additional file 2.** Hydrogen concentration in gas phase of reactors.

**Additional file 3.** Average nucleotide identity between MAGs in this study and genomes in NCBI database (hit with 85% similarity).

**Additional file 4.** The average coverage and relative abundance of each MAG in 9 samples.

**Additional file 5.** The average RPKM and relative activity of each MAG in 9 samples.

**Additional file 6.** The correspondence among methane yield, expression of mrcA gene, overall archaeal relative abundance and activity.

**Additional file 7.** The expression level of acetate kinase, acyl-CoA synthetase and formyltetrahydrofolate synthetase genes.

**Additional file 8.** Genes used for methanogenic pathway reconstructions in five archaeal MAGs and their regulation towards external hydrogen.

**Additional file 9.** The reconstruction of eha, ehb and ech cluster in different hydrogenotrophic methanogens.

**Additional file 10.** The expression level of glycine cleavage system H protein, acetyl-CoA decarbonylase/synthase complex and glycine reductase genes.

**Additional file 11.** Gene expression profile of unclassified Bacteria sp.DTU645.

**Additional file 12.** Gene expression profile of unclassified Synergistaceae sp.DTU638.

**Additional file 13.** Gene expression profile of Coprothermobacter proteolyticus DTU632.

**Additional file 14.** Average expression level of each KEEG module.

## Data Availability

The datasets generated and/or analysed during the current study are available in the sequence read archive (SRA, https://www.ncbi.nlm.nih.gov/sra) and Genomes OnLine Database (GOLD, https://gold.jgi.doe.gov/) and Integrated Microbial Genomes & Microbiomes (IMG, https://img.jgi.doe.gov/). Additional data are all provided as Supplementary Datasets in Additional files.

## Abbreviations

*ack*: Acetate kinase gene
*acs*: Acyl-CoA synthetase gene
AMP-ACS: AMP-forming acyl-CoA synthetase pathway
*Cdh*: Acetyl-CoA decarbonylase/synthase
*coo*: Carbon monoxide dehydrogenase gene
*Ech*: Energy converting hydrogenase
*Ehb*: Energy-converting hydrogenase B
*Fpo*: F_420_H_2_ dehydrogenase
*Frh*: Coenzyme F_420_-reducing hydrogenase
*gcv*: Glycine decarboxylase genes
*grd*: Glycine reductase gene
*hdr*: Membrane-bound heterodisulfide reductase
MAG: Metagenome assembled genome
*mcrA*: Methyl coenzyme M reductase gene
*mta*, *mtb*: Coenzyme M methyltransferase genes
PTA-ACKA: Phosphotransacetylase-acetate kinase pathway
RPKM: Reads per kilobase of exon model per million mapped reads
*Vho*: Methanophenazine-reducing hydrogenase,
WL: Wood-Ljungdahl
