# Supplementary figures and images for: Metabolic dependencies govern microbial syntrophies during methanogenesis in an anaerobic digestion ecosystem
Source: Microbiome. 2020 Feb 15;8:22. doi: 10.1186/s40168-019-0780-9 (PMC7024554; doi:10.1186/s40168-019-0780-9)

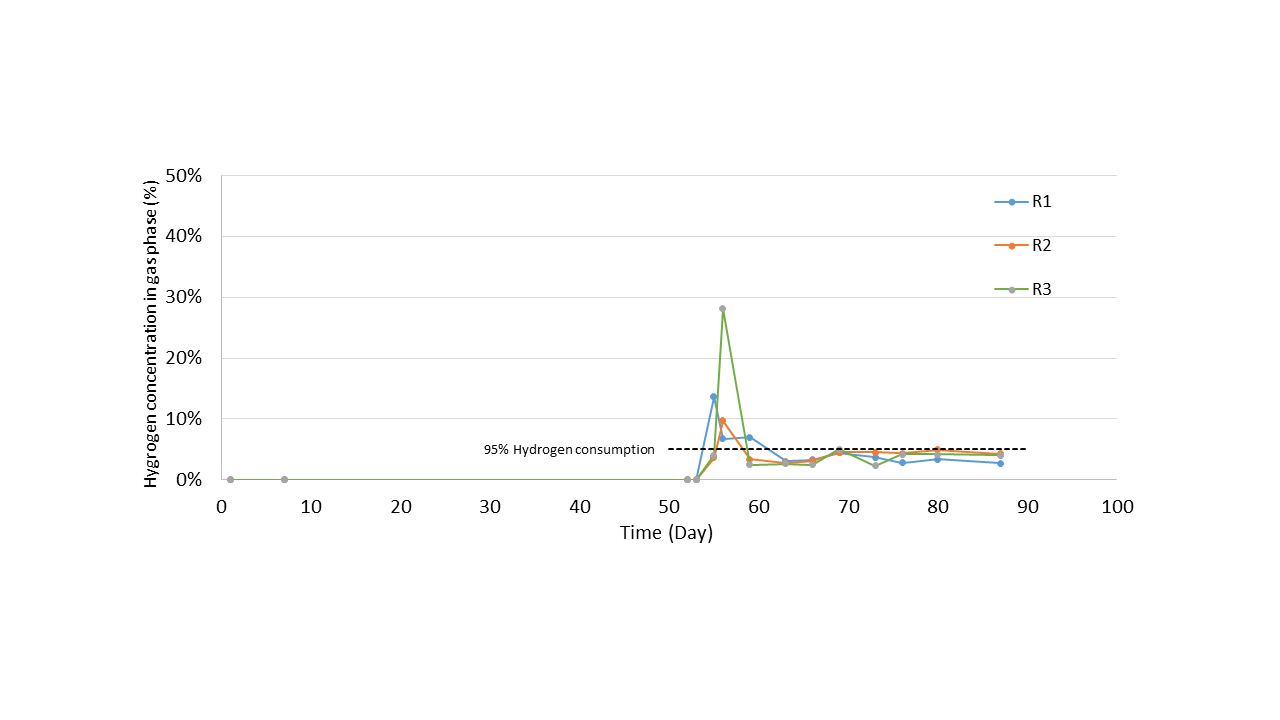

Supplement: Supplementary file 3 — Additional file 2. Hydrogen concentration in gas phase of reactors. [file 40168_2019_780_MOESM2_ESM.tif]
